# DNA Metabarcoding Reveals the Fungal Community on the Surface of Lonicerae Japonicae Flos, an Edible and Medicinal Herb

**DOI:** 10.3390/ijms242015081

**Published:** 2023-10-11

**Authors:** Yujie Dao, Jingsheng Yu, Meihua Yang, Jianping Han, Chune Fan, Xiaohui Pang

**Affiliations:** Institute of Medicinal Plant Development, Chinese Academy of Medical Sciences & Peking Union Medical College, Beijing 100193, China; yjdao@hotmail.com (Y.D.); yujsimplad@hotmail.com (J.Y.); mhyang@implad.ac.cn (M.Y.); jphan@implad.ac.cn (J.H.); fce8268958@163.com (C.F.)

**Keywords:** Lonicerae Japonicae Flos, herbal tea and medicine, fungi, DNA metabarcoding, collection area, processing method

## Abstract

Lonicerae Japonicae Flos (LJF) has been globally applied as an herbal medicine and tea. A number of reports recently revealed fungal and mycotoxin contamination in medicinal herbs. It is essential to analyze the fungal community in LJF to provide an early warning for supervision. In this study, the fungal community in LJF samples was identified through DNA metabarcoding. A total of 18 LJF samples were collected and divided based on the collection areas and processing methods. The results indicated that Ascomycota was the dominant phylum. At the genus level, *Rhizopus* was the most abundant, followed by *Erysiphe* and *Fusarium*. Ten pathogenic fungi were detected among the 41 identified species. Moreover, *Rhizopus*, *Fusarium*, and *Aspergillus* had lower relative abundances in LJF samples under oven drying than under other processing methods. This work is expected to provide comprehensive knowledge of the fungal community in LJF and a theoretical reference for enhanced processing methods in practical manufacturing.

## 1. Introduction

Lonicerae Japonicae Flos (LJF), a traditional Chinese medicine, is dried from flower buds or newly boomed flowers of *Lonicera japonica* Thunb. [1,2,3]. LJF was first recorded in *Shen Nong Ben Cao Jing* for its medicinal effect on alleviating fever and detoxification in the East Han Dynasty [2]. In modern pharmacology, LJF was first recorded in the Pharmacopoeia of the People’s Republic of China in 1995, and now, it has been used in more than 500 prescription drugs [4,5]. According to recent reports, LJF has played an important role in the production of anticancer and anti-COVID-19 drugs globally [6,7]. In addition, LJF is considered an herbal tea for its function of clearing away heat and toxins, and it has received considerable popularity for a long time in East Asia [8]. Traditionally, LJF mainly grows in Shandong, Hebei, and Henan provinces in China [3,4,9,10]. Nowadays, with the increasing demand of markets, LJF-cultivated locations have spread to Southwest China, including Sichuan and Guangxi provinces [11,12]. The processing of LJF in the market primarily includes two methods (oven drying and shade drying). Few studies have compared the effect of the production areas and the processing methods on the fungal community in LJF.

Recent studies reported that herbs could be naturally contaminated by fungi in various procedures, including cultivation, processing, transporting, storage, and marketing [13,14,15]. Le et al. detected 153 fungi in medicinal plants collected from Vietnam and identified these microorganisms in seven genera, mainly including *Alternaria*, *Fusarium*, and *Penicillium* [16]. In the Kingdom of Saudi Arabia, Al-Hindi et al. analyzed the fungal contamination in 50 herbal samples collected from the local market and indicated that *Aspergillus*, *Penicillium*, *Fusarium*, and *Rhizopus* were the main contaminated genera [17]. In 2016, 187 fungi were isolated by Aiko et al. from 58 out of 63 medicinal herb samples, and 28 fungal strains were found to be toxigenic [18]. Moreover, Zheng et al. (2017) detected 126 fungi in 15 different medicinal herbs through morphology and molecular identification. Their results indicated that two species in *Penicillium* and one species in *Eurotium* were identified in three LJF samples [19]. Therefore, the safety and quality of LJF have increasingly caught public attention in recent years. With the development of DNA sequencing technology, the analysis of fungal community using next-generation sequencing tools has become increasingly acceptable [20,21]. DNA metabarcoding has shown potential in monitoring the safety of herbs by accurately detecting the overall fungal composition and diversity [22,23]. At present, this technology has been applied in the analysis of the fungal community in herbs, such as Platycladi Semen, Myristicae Semen, and Ziziphi Spinosae Semen, through amplifying the internal transcribed spacer 2 (ITS2) region [24,25,26].

In this study, DNA metabarcoding was applied to investigate the fungal community in LJF samples. On the basis of production area, the samples were divided into five groups. Moreover, the influence of different processing methods on the fungal community in LJF samples was compared. This study is expected to provide a scientific and normative method to for the early warning to supervise fungal and mycotoxin contamination in the LJF industry.

## 2. Results

### 2.1. Fungal Diversity in LJF Samples

**Figure 1:**
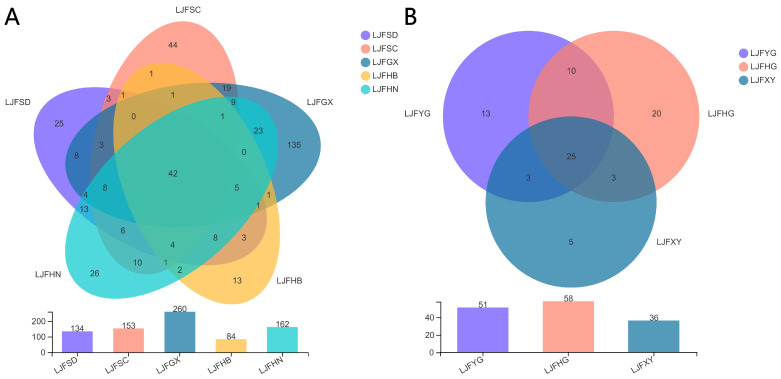
Venn analysis. (**A**) The analysis based on the production areas. (**B**) The analysis based on the processing methods.

After chimeric sequences were excluded, a total of 18 LJF samples were detected with 1,301,378 ITS2 sequences, and the average length of the sequences was 322 bp. High-quality sequences were clustered into 504 OTUs. Figure 1A vividly shows that 42 shared OTUs were detected based on production area, with 134 OTUs in LJFSD, 153 OTUs in LJFSC, 260 OTUs in LJFGX, 84 OTUs in LJFHB, and 162 OTUs in LJFHN. Of the OTUs, 25 were unique for LJFSD group, 44 were unique for LJFSC group, 135 were unique for LJFGX group, 13 were unique for LJFHB group, and 26 were unique for LJFHN group. A total of 25 shared OTUs were tested in accordance with the processing methods, with 51 OTUs in LJFYG, 58 OTUs in LJFHG, and 36 OTUs in LJFXY (Figure 1B).

Six alpha diversity indices were calculated to illustrate the community diversity, richness, evenness, and species coverage in the LJF samples (Table 1). The highest Shannon and lowest Simpson indices were observed in LJFSC1, illustrating that the sample had the highest fungal diversity among the 18 LJF samples. LJFHB3 had the lowest Shannon and highest Simpson metrices, indicating that the diversity in LJFHB3 was lower than in other LJF samples. The ACE and Chao 1 indices of LJFHB3 were lowest, indicating the lowest fungal richness. LJFGX2 had the highest ACE and Chao1 metrices, representing the richest microbial abundance in the 18 samples. The result of Good’s coverage in the 18 LJF samples suggested that the fungal community of all samples was sufficiently estimated (>99.9%). In addition, the Shannoneven index of LJFSC1 was higher than those of other samples, illustrating that the highest microbiome evenness was observed in this sample.

### 2.2. Fungal Composition in LJF Samples

**Figure 2:**
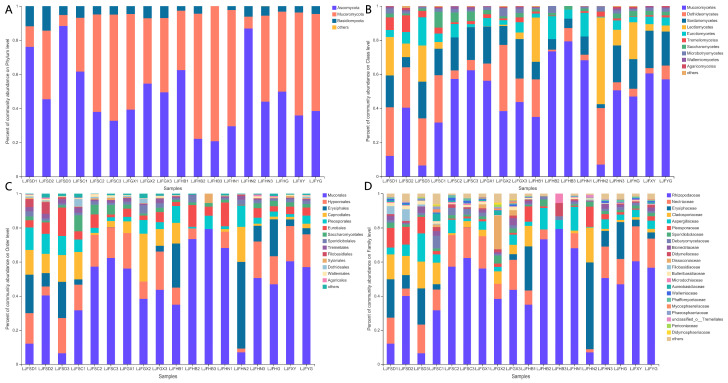
Percentage of community abundance at the phylum (**A**), class (**B**), order (**C**), and family (**D**) levels in LJF samples.

**Figure 3:**
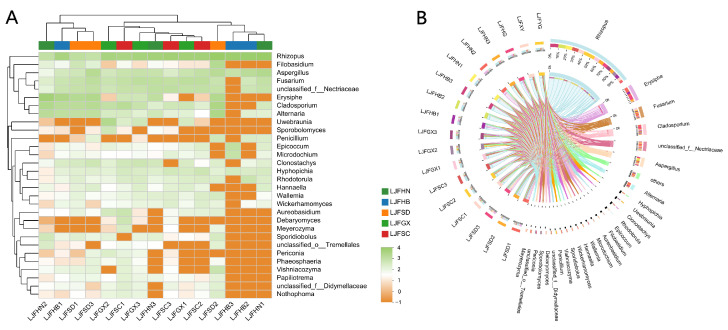
Fungal composition in LJF samples. (**A**) Heatmap of the top 30 abundant genera. (The lower the abundance of the genus, the darker the orange color; the higher the abundance of the genus, the darker the green color). (**B**) Data were visualized using Circos.

A total of 504 OTUs were obtained from the 18 LJF samples, and the distribution is listed in Supplementary Table S1. Three main phyla were detected through taxonomical classification, including Ascomycota, Mucoromycota, and Basidiomycota (Figure 2A). Ascomycota was the dominant phylum among the fungal phyla, accounting for 20.77–88.41%, followed by Mucoromycota (6.40–79.23%) and Basidiomycota (0–14.34%). At the class level, 23 classes were identified in the LJF samples. Mucoromycetes, Dothideomycetes, Sordariomycetes, Leotiomucetes, and Eurotiomycetes were the predominant classes, with relative abundances of 6.40–79.23%, 0.95–38.84%, 2.30–23.12%, 0–50.87%, and 0.41–16.55%, respectively (Figure 2B). Further taxonomic analysis at the order level showed that 72 fungal orders were identified. Mucorales had the highest abundance of 6.40–79.23%, followed by Hypocreales (0–22.46%), Erysiphales (0–50.85%), and Capnodiales (0–28.08%) (Figure 2C). The taxonomical classification showed that 166 families were detected. The relative abundance of Rhizopodaceae (6.40–79.23%) was highest, followed by Nectriaceae (0–20.49%), Erysiphaceae (0–50.85%), Cladosporiaceae (0–20.48%), and Aspergillaceae (0.36–16.54%). Rhizopodaceae and Aspergillaceae were almost distributed in the 18 LJF samples, while Nectriaceae and Cladosporiaceae could be hardly detected in LJFHB3. In addition, Erysiphaceae was not detected in LJFSC2, LJFSC3, LJFGX1, LJFGX2, LJFHB2, LJFHB3, and LJFHN1, and the relative abundance of the family in LJFHN2 was higher than in other samples (Figure 2D). The heatmap and Circos image showed the top 30 abundant genera among 274 genera detected in the 18 LJF samples (Figure 3). *Rhizopus* and *Aspergillus* were evenly distributed in each LJF sample, and they may produce important mycotoxins under proper conditions (Figure 3B). *Fusarium* was distributed in all samples except in LJFHB3. Moreover, the relative abundance of *Penicillium,* an important toxigenic fungal genus, in LJFSD2, LJFSD3, LJFHB2, and LJFHN1 was much higher than in other samples.

At the species level, 41 OTUs were accurately identified, and the remaining could be resolved at the genus or higher level via manual BLAST verification on the basis of the 100% sequence identity standard. Ten toxigenic and pathogenic fungi were detected, including *Candida tropicalis* (distributed in LJFGX2 and LJFHN2), *C. parapsilosis* (distributed in LJFHB1, LJFHN2, LJFHN3, LJFSC1, and LJFSD2), *C. sake* (distributed in LJFHN2 and LJFSD1), *Malassezia restricta* (distributed in LJFGX3, LJFHN1, LJFHN2, LJFSC1, LJFSC3, LJFSD1, LJFSD2, LJFSD3, LJFHG, and LJFYG), *M. sympodialis* (distributed in LJFHG), *Kodamaea ohmeri* (distributed in LJFHN2), *Lodderomyces elongisporus* (distributed in LJFSC1), *Schizophyllum commune* (distributed in LJFGX2, LJFHB1, LJFHN3, LJFSD2, LJFHG, LJFXY, and LJFYG), *Wallemia sebi* (distributed in LJFGX2, LJFHN2, LJFXY, and LJFYG), and *Mucor circinelloides* (distributed in LJFGX2, LJFGX3, LJFHB1, LJFHN2, LJFSC3, LJFHG, and LJFYG).

### 2.3. Fungal Comparison in LJF Samples from Five Production Areas

**Figure 4:**
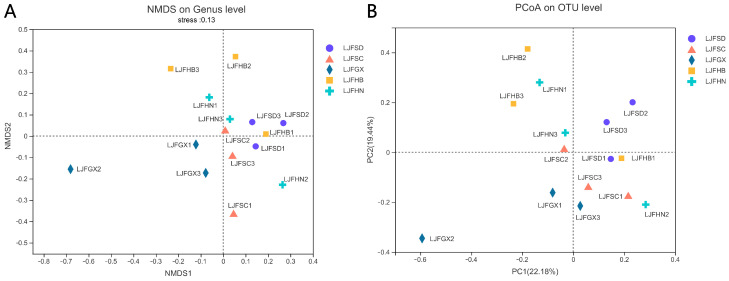
Comparison of fungal community based on the production areas. (**A**) PcoA analysis conducted at the genus level. (**B**) NMDS diagram estimated at the OTU level.

Alpha-diversity analysis showed that the LJFHB group had the lowest Shannon index and the highest Simpson index. The ACE and Chao 1 indices of the LJFHB group were lower than those of the other groups. These results illustrated that the fungal richness and diversity in the LJFHB group were lowest among the five groups. The LJFSD group had higher Shannon and Shannoneven and lower Simpson indices, indicating that the fungal diversity was larger than others. The Bar diagram demonstrated that Mucorales in the LJFSD group was lowest among the five groups at the order level. At the family level, the LJFSD group had a higher relative abundance of Pleosporaceae compared to the others. Lefse analysis was performed to compare the differences in fungal community among five LJF groups at various levels, ranging from the phylum level to genus level (Figure S1A,B). At the family level, Dissoconiaceae, Tremellaceae, and Sporocadaceae in the LJFGX group were significantly higher than those in the other groups (*p* < 0.05). The relative abundances of *Naganishia* and *Trichoderma* in the LJFSD group were remarkably more enriched at the genus level (*p* < 0.05). The NMDS analysis, conducted at the genus level, based on the QIIME calculation illustrated the similarity in the fungal composition in five LJF groups (Figure 4A). The stress index indicated that the analysis could be greatly convincing (stress < 0.2). The samples from the LJFHB group and LJFHN groups were close to the samples from the LJFSD group, indicating that these compositions were similar. The LJFGX and LJFSC group could be significantly distinguished with the LJFHB, LJFSD, and LJFHN groups. The result of PcoA analysis, which was conducted at the OTU level, similarly showed that the LJFGX and LJFSC groups varied from the others (Figure 4B).

### 2.4. Fungal Comparison in LJF Samples by Using Three Processing Methods

**Figure 5:**
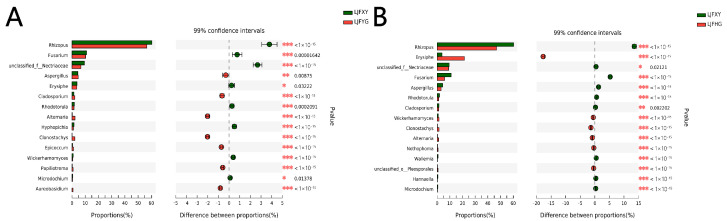
Significant difference of LJF samples based on processing method. (* *p* ≤ 0.05, ** *p* ≤ 0.01, *** *p* ≤ 0.001). (**A**) The comparison between LJFXY and LJFYG. (**B**) The comparison between LJFXY and LJFHG.

The Shannon and Simpson metrices in LJFYG and LJFHG indicated a higher diversity than that in LJFXY. The fungi were richer in LJFYG and LJFHG than in LJFXY, as revealed by the higher indices of ACE and Chao1. The difference between LJFXY and LJFYG was determined at 99% confidence intervals, as shown in Figure 5A. At the genus level, the relative abundances of *Rhizopus* and *Fusarium* in LJFXY were significantly higher than those in the LJFYG. After being processed, the LJF samples had more *Aspergillus* and *Cladosporium*, from 4.39% to 4.70% and 1.47% to 2.13%, respectively. In addition, Figure 5B shows the comparison of LJFXY with LJFHG at the genus level. The relative abundances of *Rhizopus* and *Fusarium* significantly decreased after being dried in the oven, similar to the processing method wherein samples were dried in the shade. The relative abundance of *Aspergillus* and *Cladosporium* in LJFHG was notably lower than that in LJFXY.

### 2.5. Fungal Co-Occurrence Analysis in LJF Samples

The interaction of fungi at the genus level was studied via co-occurrence analysis to reveal the microbial diversity of LJF samples (Figure S2). A total of 18 positive and 4 negative correlations were identified among the top 20 detected genera from two phyla, namely Ascomycota and Basidiomycota. The wider the line was, the closer the correlation between the genera was. The correlation between Cladosporium and Alternaria was closer than with *Erysiphe*, *Sporidiobolus*, *Vishniacozyma*, and *Filobasidium*. *Hyphopichia* displayed a negative correlation with *Alternaria*, *Cladosporium*, *Erysiphe*, and *Sporidiobolus*. *Fusarium* was positively correlated with *Clonostachys*. *Wallemia* exhibited a positive interaction with *Vishniacozyma* and *Filobasidium*.

## 3. Discussion

### 3.1. Fungal Contaminations in LJF Samples

LJF, as an herbal tea, is easily subjected to various fungal contaminants during planting, harvesting, processing, packaging, transportation, and storage [19]. Tea has been consumed as an infusion or a decoction all over the world for thousands of years, and it is filled with soluble and insoluble ingredients. These ingredients may include a number of contaminants, which pose a potential dominant health hazard for humans [27]. Fungi, as some of the contaminants in tea, have been studied in recent years. In 2020, Reinholds et al. revealed that 87% of tea samples were contaminated by 32 fungal species, in which five *Aspergillus* spp. and one *Penicillium* spp. were predominant [28]. Pakshir et al. investigated the fungal contamination in 45 black teas and 15 green teas collected from different brands. The result showed that 89% of black tea samples were contaminated by *Aspergillus* (66.7%), *Penicillium* (35.6%), *Mocur* (20%), and yeast (6.7%), while each green tea sample showed yeast (66.7%), *Aspergillus* (60%), *Mocur* (46.7%), *Penicillium* (46.7%), and *Fusarium* (13.3%) [8]. Wang et al. observed that *Fusarium* was the dominant genus in samples collected from the subtropical tea plantations of China [29]. *Aspergillus*, *Fusarium*, *Penicillium*, and yeast were the main fungi detected from tea samples in previous studies. In 2020, Liu et al. demonstrated the high abundance of *Aspergillus*, *Penicillium*, *Xanthomonas*, *Microcystis*, *Talaromyces*, and *Erysiphe* in five LJF samples on the basis of ITS sequencing [30]. In the present study, the fungal community in 18 LJF samples was investigated through DNA metabarcoding on the basis of ITS2 sequences. *Aspergillus*, *Fusarium*, and *Penicillium* were detected in 18 LJF samples. The relative abundance of *Fusarium* accounted for 0–11.28%, followed by *Aspergillus* (0.36–15.62%) and *Penicillium* (0–1.49%). Much attention has been paid to *Fusarium* as the primary genus detected in Chinese subtropical tea plantations [31]. *Aspergillus* and *Penicillium*, which could consequently affect human and animal health, have been reported for years to have toxigenic characteristics.

Ten potential pathogenic fungi belonging to *Candida*, *Malassezia*, *Kodamaea*, *Lodderomyces*, *Schizophyllum*, *Wallemia*, and *Mucor* were detected via manual BLAST based on 100% accuracy. *C. tropicalis* could cause human diseases under proper conditions, such as bloodstream infections and candidaemia [32,33]. *C. parapsilosis*, *K. ohmeri*, and *L. elongisporus*, important nosocomial pathogens, may infect weak patients in hospitals and even threaten life [34,35,36]. *M. restricta*, *M. sympodialis*, and *W. sebi* were related to bowel disease, dermatological disorders, and systemic infections [37,38]. *M. circinelloides* could contribute to thrombosis and fatal mucormycosis [39]. *S. commune* has the ability to cause serious infection, such as sinusitis, in patients who are ill [40]. As a result, these pathogenic fungi may potentially threaten the safety and quality of LJF products and public health. This work could play a role as an early warning to supervise the fungal community in LJF samples to guarantee human health.

### 3.2. Effect of Processing Methods in LJF Samples

Tea, an everyday drink for some people, can be classified into several categories, including black, white, and green tea, on the basis of different processing methods. During the processing procedure, the fungal community exhibited some remarkable differences in various tea categories [41]. Fu brick tea, a post-fermented tea, could be greatly influenced by the microbial change during the manufacturing process procedure, as reported by Li et al. in 2017. The research showed that *Aspergillus* was the dominant genus among the whole detected genera during manufacturing. The abundance of *Aspergillus* significantly increased during fermentation and even accounted for 99.99% at the end of the fermentation stage of Fu brick tea [42]. In 2020, the fungal community of Cassiae Semen, a roasted tea, was investigated on the basis of the processing methods by Guo et al., involving raw and roasted materials [43]. The result indicated that the *Penicillium* in roasted samples was much more distributed than the raw materials were, and the relative abundances of *Aspergillus*, *Cladosporium*, *Alternaria*, and *Rhizopus* were significantly higher in raw tea. In 2021, Tong et al. detected the microbial populations in green tea and black tea samples, and in the leaves of the *Camellia sinensis*, in accordance with different processing methods, including fresh, dry, and withering samples [44]. *Alternaria*, *Cladosporium*, *Aspergillus*, and *Candida* were less abundant in dry and withering samples than in fresh samples. After the samples were dried and withered, the relative abundance of *Debaryomyces* significantly increased. LJF, as one of the simplest manufacturing teas, was considered as the least contaminated tea group during the procedure [41]. Previous studies illustrated that the desiccation stage could significantly decrease the abundance of *Aspergillus*, *Cladosporium*, *Alternaria*, and *Rhizopus* in the tea samples. In the present study, various degrees of fungi contaminated each LJF sample. LJFHG that was dried in the oven had a lower abundance of *Rhizopus*, *Aspergillus*, and *Cladosporium* than LJFXY (fresh LJF sample). *Aspergillus* and *Cladosporium* were more abundant in LJFYG (shade-dried) than in LJFHG. The LJF samples that were dried in the oven had less pathogenic fungi than those that underwent other processing methods. Therefore, LJF should be timely dried in the oven to control the fungal contamination during manufacturing. Moreover, our study indicated that production area also had an impact on the fungal community in LJF samples. Samples from Guangxi and Sichuan, which are at similar latitudes, showed little difference in their fungal composition. The fungal composition in samples from Guangxi and Sichuan was significantly different from those in samples from Hebei, Shandong, and Henan. The above similarities or differences may be related to the climatic conditions of the producing areas.

## 4. Materials and Methods

### 4.1. Sample Collection

A total of 18 LJF samples were collected from different production areas in China. The 15 dried samples from five provinces (Shandong, Hebei, Henan, Guangxi, and Sichuan) were divided into five groups on the basis of production area, namely, LJFSD, LJFHB, LJFHN, LJFGX, and LJFSC. The other samples from Beijing were classified into three groups on the basis of processing method, namely, LJFXY, LJFHG, and LJFYG. LJFXY was not processed. The materials of LJFHG were dried in the oven for 10 h at a temperature of 55 °C ± 5 °C, while those of LJFYG were dried in the shade for 15 days. Table 2 lists the information of 18 LJF samples.

### 4.2. Total DNA Extraction and Polymerase Chain Reaction (PCR) Amplification

Approximately 0.91 g of LJF samples was weighed and transferred into a 50 mL sterilized centrifuge tube, and 25 mL of 1×PBS buffer (Beijing Solarbio Science & Technology Co., Ltd., China) was added. We then shook the tube with the vortex mixer for five minutes and filtered the solution using four layers of sterile gauze. Then, the solution was centrifugated for 28 min to collect the fungal strains (Centrifuge 5430 R, Eppendorf AG, Hamburg, Germany). And we extracted the total DNA by following the manufacturer’s instructions of EZNA^®^ soil DNA kit (Omega Bio-tek., Inc., Norcross, GA, USA). The sequences were amplified by targeting the ITS2 region using the designed primer pairs ITS3 (5′-GCATCGATGAAGAACGCAGC-3′) and ITS4 (5′-TCCTCCGCTTATTGATATGC-3′) [45]. The PCR condition was performed as follows: initial denaturation for 5 min at 94 °C; 40 cycles of denaturation for 30 s at 94 °C, annealing for 30 s at 56 °C and then elongation for 45 s at 72 °C; and final extension for 10 min at 72 °C. Each sample was amplified three times, which were pooled to reduce the PCR bias. Agarose (2%, 447 *W*/*V*) gel electrophoresis and DNA extraction kit were used to verify and purify the desired products (Axygen, Union City, CA, USA).

### 4.3. DNA Metabarcoding and Data Analysis

The amplified ITS2 sequences were subjected to Illumina Miseq PE300 platform (Illumina, San Diego, CA, USA). The raw amplifications were uploaded to the National Center for Biotechnology Information Sequence Read Archive database with accession numbers SAMN24255458–SAMN24255475. The raw reads were truncated at any site (a minimum 10 bp overlap) to receive a quality score of at least 20 over a 50 bp sliding window by using Fastp software (v. 0.19.6 https://github.com/OpenGene/fastp, accessed on 9 October 2021). And all the trimmed sequences were clustered into operational taxonomic units (OTUs) with 97% similarity using UPARSE (version 7.0.1090, http://www.drive5.com/uparse/, accessed on 9 October 2021) and USEARCH (version 8.1.1861, http://www.Drive5.Com/Usearch/, accessed on 9 October 2021) [46]. The OTUs were annotated at various levels ranging from genus to phylum level based on taxonomical classification in accordance with the UNITE database. Then, the OTUs were checked via manual basic local alignment search tool (BLAST) search in accordance with the International Nucleotide Sequence Database Collaboration with 100% species similarity. Alpha diversity was evaluated through Mothur (v. 1.30.2 https://www.mothur.org/wiki/Download_mothur, accessed on 9 October 2021) by calculating six indices, including Chao1, ACE, Good ’s coverage, Shannon, Simpson, and Shannoneven [47]. Higher Shannon and lower Simpson metrices reflect higher fungal diversity in samples. Higher ACE and Chao 1 indices represent richer fungal community. Good’s coverage index indicates the depth of sequences detected in the samples. Venn diagram, bar map, and heatmap were created using R software (version 3.3.1), and Circos image was created in Circos software (version 0.67-7, http://circos.ca/, accessed on 9 October 2021). Beta diversity was assessed to analyze the differences in fungal community at the genus level through non-metric multidimensional scaling (NMDS) analysis (version 330 1.9.1 http://qiime.org/install/index.html, accessed on 9 October 2021). The similarity and diversity in different groups were investigated at the OTU level using principal coordinate analysis (PCoA) through the Bray–Curtis distance matrix. Linear discriminant analysis effect size (LEfSe) analysis was conducted to compare the significant differences from phylum level to genus level (http://huttenhower.sph.harvard.edu/galaxy/root?tool_id=lefse_upload, accessed on 9 October 2021). Statistical difference was analyzed via chi-square test to compare the difference in fungal composition. Network analysis was performed using NetworkX software to illustrate the correlation among the top 20 genera [48].

## 5. Conclusions

In this study, the fungal community diversity and composition of 18 LJF samples were surveyed using ITS2 amplicon sequencing. The results indicated that *Rhizopus*, *Erysiphe*, and *Fusarium* were the dominant fungi at the genus level. In addition, *Rhizopus*, *Fusarium*, and *Aspergillus* had lower relative abundances in LJF samples under oven drying than under other processing methods. DNA metabarcoding could effectively detect ten potential toxigenic fungi and human pathogenic yeast in edible-medicinal herbs, assisting in evaluating food safety. The method will continually be applied to explore the fungal structure in more herbal medicine and tea for future research.

## Figures and Tables

**Table 1:** Alpha diversity of the fungal community in LJF samples.

Sample	Shannon	Simpson	ACE	Chao1	Good’s Coverage	Shannoneven
LJFSD1	2.76	0.10	80.74	80.33	1	0.64
LJFSD2	2.62	0.16	78.62	78.00	1	0.60
LJFSD3	2.71	0.10	73.74	73.50	1	0.63
LJFHB1	2.21	0.18	76.68	76.50	1	0.51
LJFHB2	1.50	0.41	0.00	20.00	1	0.50
LJFHB3	1.16	0.47	8.00	8.00	1	0.56
LJFHN1	1.74	0.36	45.89	38.00	1	0.48
LJFHN2	1.70	0.32	163.43	166.40	1	0.34
LJFHN3	2.21	0.21	52.00	48.00	1	0.58
LJFGX1	2.20	0.25	65.78	64.33	1	0.53
LJFGX2	2.73	0.16	217.54	217.50	1	0.51
LJFGX3	2.73	0.16	93.27	93.00	1	0.60
LJFSC1	3.17	0.10	123.51	123.50	1	0.66
LJFSC2	2.05	0.26	47.03	46.50	1	0.54
LJFSC3	2.01	0.30	65.27	65.00	1	0.48
LJFYG	2.19	0.25	52.05	52.00	1	0.56
LJFHG	2.12	0.22	58.48	58.00	1	0.52
LJFXY	1.93	0.28	46.00	39.00	1	0.54

**Table 2:** Voucher number and GenBank accession numbers of LJF samples in this study.

Voucher No.	Sampling Location	Group 1	Group 2	Collection Time	GenBank Accession No.
LJFSD1	Shandong	LJFSD	/	July 2021	SAMN24255458
LJFSD2	Shandong	LJFSD	/	July 2021	SAMN24255459
LJFSD3	Shandong	LJFSD	/	July 2021	SAMN24255460
LJFHB1	Hebei	LJFHB	/	July 2021	SAMN24255461
LJFHB2	Hebei	LJFHB	/	July 2021	SAMN24255462
LJFHB3	Hebei	LJFHB	/	July 2021	SAMN24255463
LJFHN1	Henan	LJFHN	/	July 2021	SAMN24255464
LJFHN2	Henan	LJFHN	/	July 2021	SAMN24255465
LJFHN3	Henan	LJFHN	/	July 2021	SAMN24255466
LJFGX1	Guangxi	LJFGX	/	July 2021	SAMN24255467
LJFGX2	Guangxi	LJFGX	/	July 2021	SAMN24255468
LJFGX3	Guangxi	LJFGX	/	July 2021	SAMN24255469
LJFSC1	Sichuan	LJFSC	/	July 2021	SAMN24255470
LJFSC2	Sichuan	LJFSC	/	July 2021	SAMN24255471
LJFSC3	Sichuan	LJFSC	/	July 2021	SAMN24255472
LJFYG	Beijing	/	LJFYG	July 2021	SAMN24255473
LJFHG	Beijing	/	LJFHG	July 2021	SAMN24255474
LJFXY	Beijing	/	LJFXY	July 2021	SAMN24255475

## Data Availability

The data presented in this study are available on request.

## Supplementary Materials

The following supporting information can be downloaded at https://www.mdpi.com/article/10.3390/ijms242015081/s1.
