# Supplementary figures and images for: DNA Metabarcoding Reveals the Fungal Community on the Surface of Lonicerae Japonicae Flos, an Edible and Medicinal Herb
Source: Int J Mol Sci. 2023 Oct 11;24(20):15081. doi: 10.3390/ijms242015081 (PMC10606453; doi:10.3390/ijms242015081)

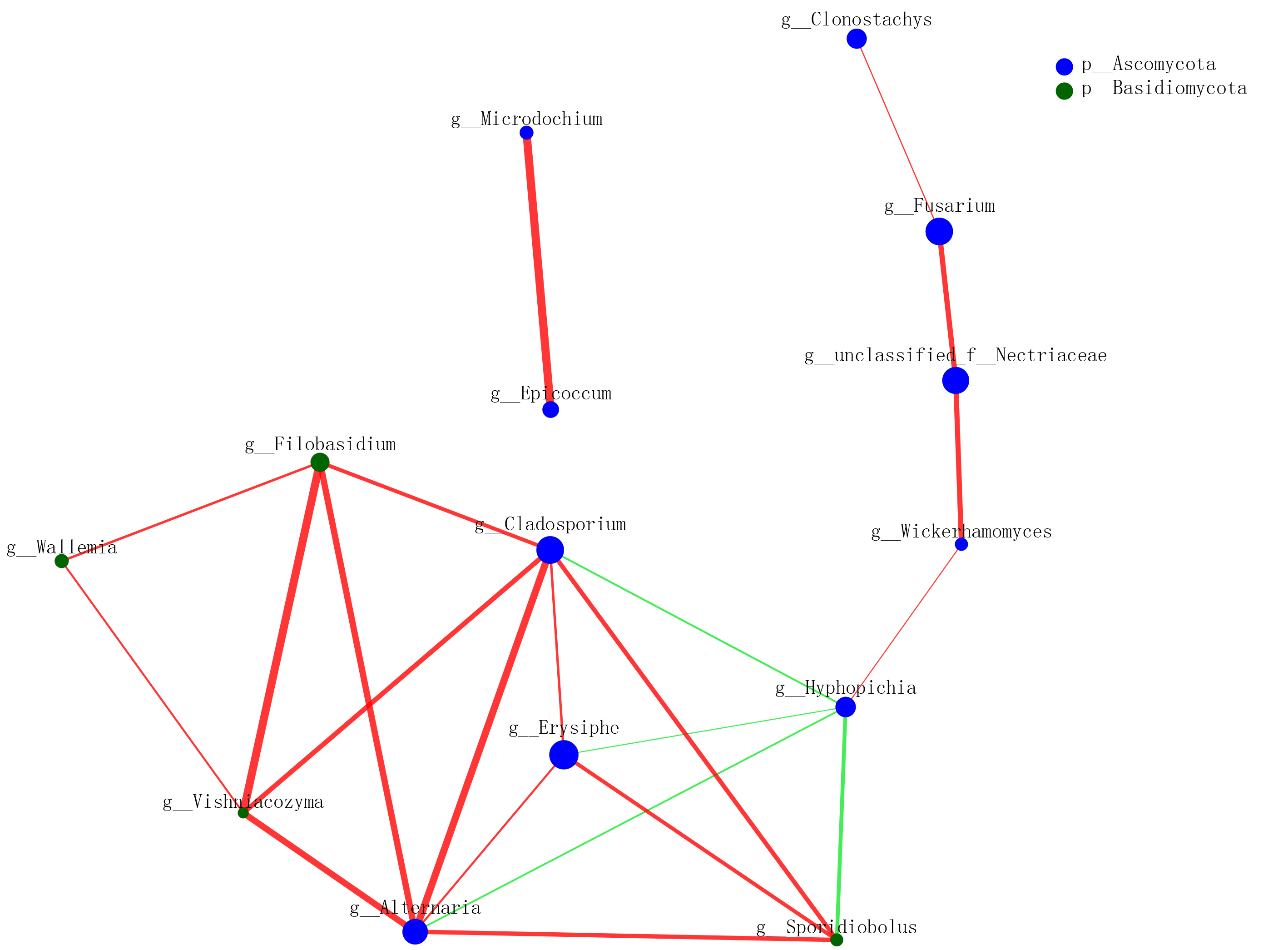

Supplement: Supplementary file 1 [file ijms-24-15081-s001.zip › Supplemental Figure 2..tif]

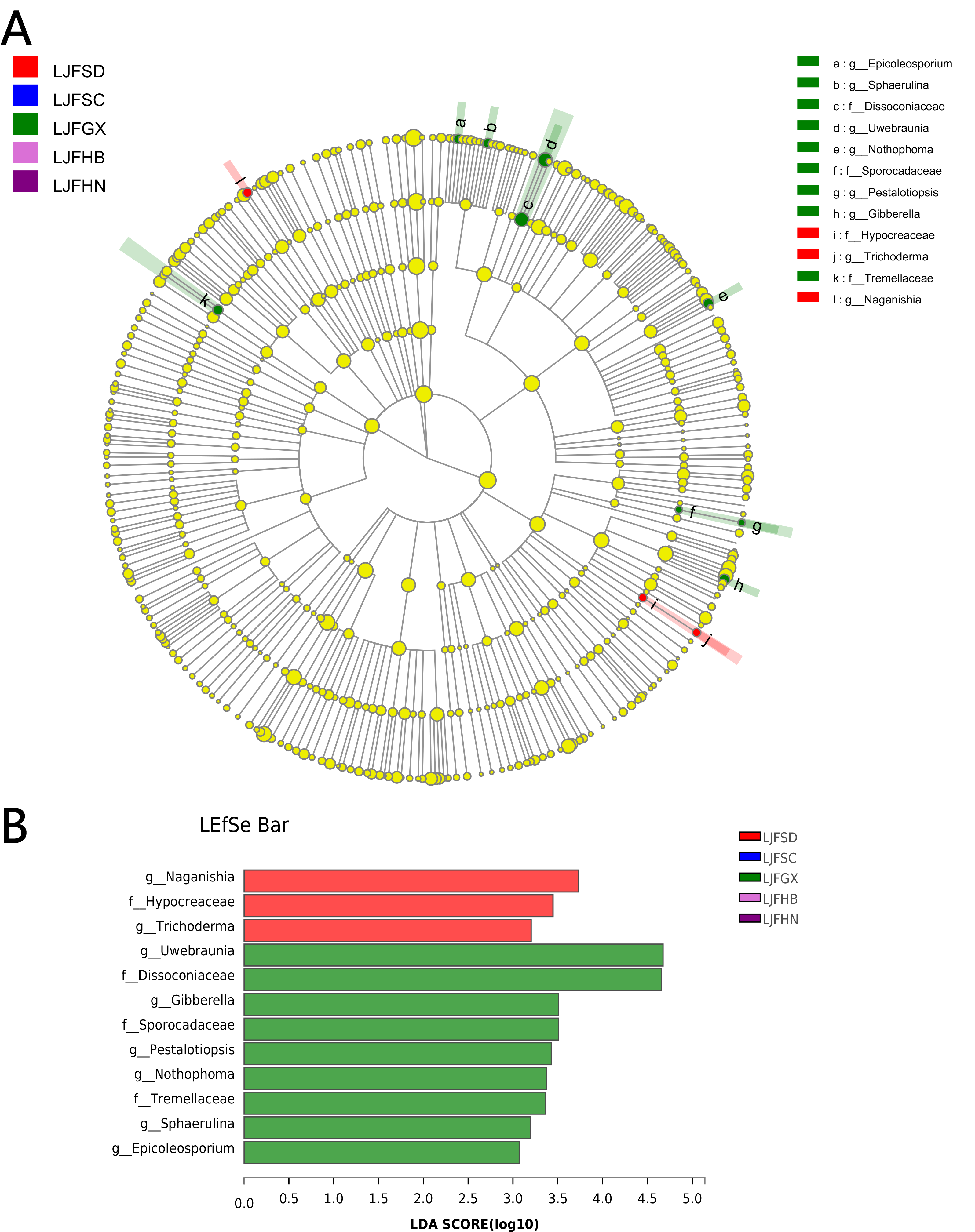

Supplement: Supplementary file 1 [file ijms-24-15081-s001.zip › Supplemental Figure 1..tif]
